# *In memoriam* Thomas Gebel

**DOI:** 10.1007/s00204-023-03473-9

**Published:** 2023-03-14

**Authors:** Jan G. Hengstler, Hermann Bolt, Uwe Heinrich, Andrea Hartwig, Robert Landsiedel

**Affiliations:** IfADo Leibniz Research Centre for Working Environment and Human Factors at the Technical University of Dortmund, Mainz, Germany


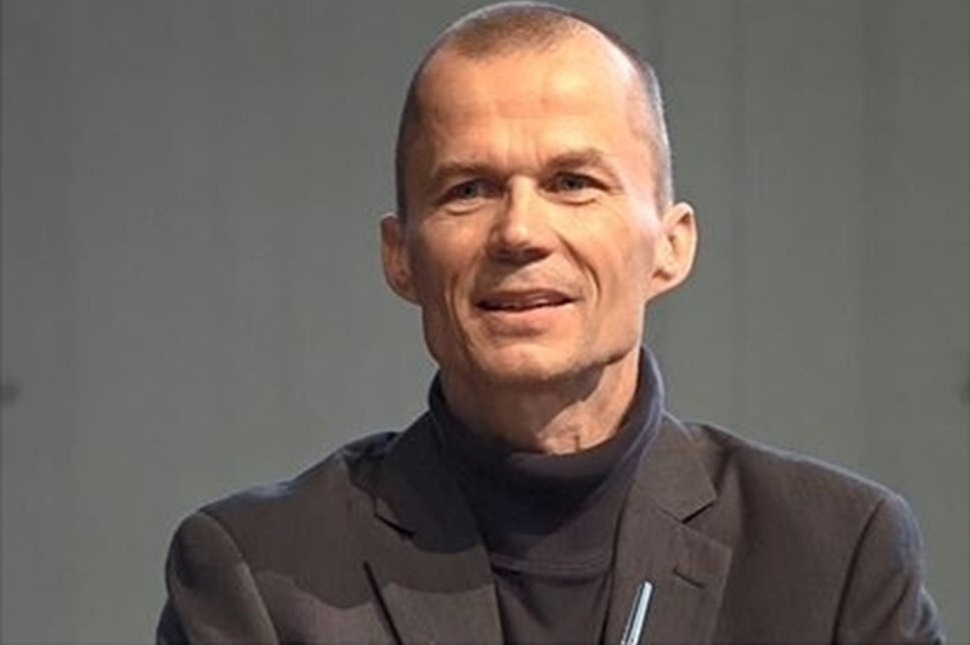


We are saddened by the passing of Prof. Tom Gebel, Scientific Director at the Federal Institute of Occupational Safety and Health, on January 23rd, 2023.

Tom Gebel was born in Düsseldorf in 1965. He studied biology at the universities of Aachen and Marburg, where he wrote his diploma thesis on carbonyl reduction of metyrapone, a drug used to diagnose adrenal insufficiency. From 1991 to 1993, he worked on his Ph.D. thesis on the mechanisms of peroxisome proliferation at the Institute of Toxicology in Mainz under the mentorship of Professor Franz Oesch. He obtained his *venia legendi* in 1999 at the Medical Faculty in Göttingen and was appointed a professorship at the Faculty of Chemistry and Chemical Biology in Dortmund in 2010. He joined the Federal Institute of Occupational Safety and Health in 2001 where he later became a Scientific Director.

Tom Gebel was a member of the Scientific Advisory Board of the German Society of Toxicology and contributed to numerous reviews on a range of applied toxicological topics, such as the categorization of manufactured nanomaterials, risk evaluation of bisphenol A, inorganic arsenic, the accumulation of lead in soils, human health hazards due to hydraulic fracturing, health hazards due to bromate and a critical discussion on the regulatory changes for endocrine disruptors and mixture toxicity. Moreover, Tom was active in the program commission and the working group on Regulatory Toxicology of the German Society of Toxicology.

Moreover, Tom Gebel served as a member of the Editorial Board of Archives of Toxicology since 2014 and was an ad hoc expert for the Permanent Senate Commission for the Investigation of Health Hazards of Chemical Compounds in the Work Area and the World Health Organization.

One of Tom Gebel’s specific scientific interests was the genotoxicity of arsenic compounds, platinum and palladium and the biomonitoring of arsenic and antimony, topics to which he contributed several well-recognized studies. Another focus of his interests was on health effects of airborne particulate matters at workplaces and deriving Occupational Exposure Limits for materials such as Diesel soot, crystalline quartz, metal and fibre dusts and nanoparticles. His great expertise in evaluating granular and fibrous particles was highly appreciated. He initiated research activities in areas where data for derivation of health-based Occupational Exposure Limits were missing. One of his recent ambitious research activities was a joint project of EU, German Federal Ministries, industry and research institutes (*NanoInVivo*), to gain a better understanding of long-term impacts of market-relevant nanomaterials.

Tom Gebel was continuously engaged to identify health risks to exposed workers and find ways to reduce these. He did so in a much appreciated, pragmatic way applying the most recent toxicological science risk assessment, acting both on the national as well as on the EU level.

Tom Gebel always impressed us with his absolute integrity as he was solely guided by his scientific acumen, even on the most controversial topics. With the passing of Tom Gebel, we have lost a remarkable scientist, a consequent and critical conversationalist, brilliant thinker, and, last but not least, a dear friend and colleague. We offer our sincere condolences to his wife and two children.

*Jan G. Hengstler, Uwe Heinrich, Hermann Bolt, Andrea Hartwig and Robert Landsiedel* for the board and members of the German Society of Toxicology.

