# Correction: *In memoriam* Thomas Gebel

**DOI:** 10.1007/s00204-023-03493-5

**Published:** 2023-04-21

**Authors:** Jan G. Hengstler, Hermann Bolt, Uwe Heinrich, Andrea Hartwig, Robert Landsiedel

**Affiliations:** 1 Leibniz Research Centre for Working Environment and Human Factors (IfADo), Technical University of Dortmund, 44139 Dortmund, Germany; 2 Medizinische Hochschule Hannover, 30625 Hannover, Germany; 3 Department of Food Chemistry and Toxicology, Institute of Applied Biosciences (IAB), Karlsruhe Institute of Technology (KIT), 76131 Karlsruhe, Germany; 4 Experimental Toxicology and Ecology, BASF SE, 67056 Ludwigshafen am Rhein, Germany

**Correction: Archives of Toxicology** 10.1007/s00204-023-03473-9

The Editorial was accidentally published with the wrong affiliations and addresses.

We apologize for this and provide the correct affiliations and addresses below.

Hengstler JG^1^✉, Bolt H^1^, Heinrich U^2^, Hartwig A^3^, Landsiedel R^4^

^1^Leibniz Research Centre for Working Environment and Human Factors (IfADo), Technical University of Dortmund, 44139 Dortmund, Germany

^2^Medizinische Hochschule Hannover, 30625 Hannover, Germany

^3^Department of Food Chemistry and Toxicology, Institute of Applied Biosciences (IAB), Karlsruhe Institute of Technology (KIT), 76131 Karlsruhe, Germany

^4^Experimental Toxicology and Ecology, BASF SE, 67056 Ludwigshafen am Rhein, Germany

✉ Jan G. Hengstler hengstler@ifado.de

